# Numerical and Experimental Analysis of Inhalation Airflow Dynamics in a Human Pharyngeal Airway

**DOI:** 10.3390/ijerph17051556

**Published:** 2020-02-28

**Authors:** Yaming Fan, Jingliang Dong, Lin Tian, Kiao Inthavong, Jiyuan Tu

**Affiliations:** 1 Indoor Environment Engineering Research Center of Fujian Province, College of Ecological Environment and Urban Construction, Fujian University of Technology, Fuzhou 350118, China; chenfanyaming@126.com; 2 School of Engineering, RMIT University, P.O. Box 71, Bundoora, VIC 3083, Australia; kiao.inthavong@rmit.edu.au (K.I.); jiyuan.tu@rmit.edu.au (J.T.)

**Keywords:** human pharynx, numerical simulation, in vitro measurements, airway collapse, obstructive sleep apnea

## Abstract

This paper presents a computational and experimental study of steady inhalation in a realistic human pharyngeal airway model. To investigate the intricate fluid dynamics inside the pharyngeal airway, the numerical predicted flow patterns are compared with in vitro measurements using Particle Image Velocimetry (PIV) approach. A structured mesh with 1.4 million cells is used with a laminar constant flow rate of 10 L/min. PIV measurements are taken in three sagittal planes which showed flow acceleration after the pharynx bend with high velocities in the posterior pharyngeal wall. Computed velocity profiles are compared with the measurements which showed generally good agreements with over-predicted velocity distributions on the anterior wall side. Secondary flow patterns on cross-sectional slices in the transverse plane revealed vortices posterior of pharynx and a pair of secondary flow vortexes due to the abrupt cross-sectional area increase. Finally, pressure and flow resistance analysis demonstrate that greatest pressure occurs in the superior half of the airway and maximum in-plane pressure variation is observed at the velo-oropharynx junction, which expects to induce a high tendency of airway collapse during inhalation. This study provides insights of the complex fluid dynamics in human pharyngeal airway and can contribute to a reliable approach to assess the probability of flow-induced airway collapse and improve the treatment of obstructive sleep apnea.

## 1. Introduction

Biomechanical modelling of the human upper airway has attracted intensive research attention for decades since it enables a better understanding of its physiology and pathophysiology. Growing interest in the pharynx is largely due to the recognition of obstructive sleep apnea (OSA) [1,2] and pollutant-related epidemiology [3,4,5]. Therefore, detailed knowledge of airflow dynamics in the pharyngeal airway is central to these issues as it underpins the mechanisms of air-structure and air-particle interactions in the upper and lower airways.

The geometry in the human pharynx is a wall-bounded airway that exhibits irregular surfaces and cross-sectional areas. From a macro perspective, its gross geometry conforms to a 90-degree bend pipe. Therefore, it is anticipated that airflow patterns inside the pharynx should produce some similar fluid dynamics found in internal bend pipes. This includes flow separation, reattachment, and secondary flow vortices (Dean vortices). In parallel with great amount of clinical studies within this field [6,7,8], multidisciplinary approaches that combines medical imaging and numerical modelling techniques have been widely adopted to investigate the interplay between physiology, mechanics and pathophysiology [9,10,11,12,13]. 

**Figure 1:**
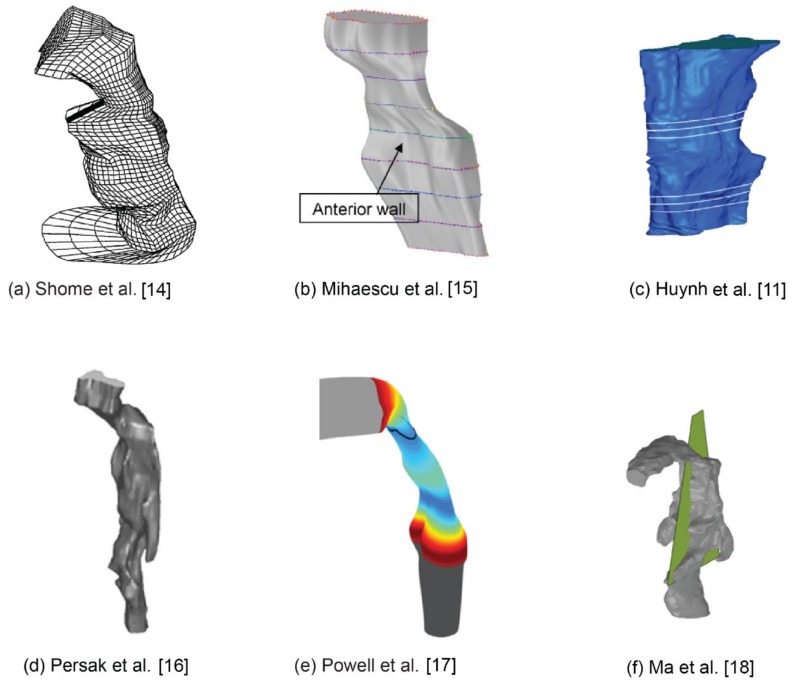
Computational models of the pharyngeal airway studied in the literature.

Some relevant numerical studies using image-based pharyngeal airway models were shown in Figure 1. Shome et al. [14] is one of the first numerical studies to investigate airflow in a three-dimensional realistic pharynx under average (24 L/min) and peak (48 L/min) inspiratory flow rate conditions. They found the *k – ε* turbulence model was appropriate for simulating the transitional flow and the pressure drop in the pharynx lies in the range 200–500 Pa. Later, Mihaescu et al. [15] compared turbulence models for flow in the pharynx concluding that the *k* - *ω* model performs better than the *k* - *ε* model. Huynh et al. [11] evaluated pharyngeal airflow in obstructive sleep apnea patients following maxillomandibular advancement surgery. They found that after surgery, the pressure gradient and maximum velocity within the pharynx decreased, suggesting an improvement to respiratory function. Persak et al. [16] developed a numerical model that investigates airflow dynamics in a deforming pharynx during a respiration cycle, which provides a more comprehensive and realistic view of airway mechanics in individual patients during tidal breathing with or at risk for OSA. Powell et al. [17] performed a comparative study between four sleep apneic subjects and four healthy controls. Velocity fields, static pressure and wall shear stress (WSS) distributions were calculated. Possible relationships deduced from their research findings are: airway zones with reduced static pressure may be more susceptible to collapse; Increased WSS and high frequency shear stress variations induced by jet impingement and reversed flow may possibly lead to tissue irritation and progressive damage; High and low frequency flow fluctuations at the airway wall due to jet flapping and recirculating separated flow are likely to induce vibrations in the compliant airway lumen. Ma et al. [18] developed a series of numerical models with different pharynx lumen size based on an anatomically accurate upper airway. This study shows the narrowing of the pharynx from lingual facts can potentially change airway flow dynamics and particle deposition. 

Although numerous studies have been carried out in this field, most of them are relying on numerical modelling approaches, while research efforts focusing on experimental measurements are relatively lacking. To date, a few experimental studies have been conducted and most of them are centered on pharyngeal compliance and its effects on airway collapse in obstructive sleep apnea. Chouly et al. [19] built a rapidly convergent-divergent geometry using a water-filled latex tube inserted perpendicular to a rigid pipe to represent a simplified pharyngeal airway, which allows airway deformation measurements under expiratory airflow conditions. Le et al. [20] conducted a similar study, but with an anatomically accurate, collapsible model of the human pharynx, which is a significant improvement compared to the previous study conducted by Chouly and coauthors [19]. Most recently, Zhao et al. [21] developed an experimental rig to investigate the effects of airway compliance on airflow dynamics during respiration by using Particle Imaging Velocimetry (PIV) technique. Their results revealed notable flow differences between undeformed case and soft-tissue case, which highlights the importance of considering wall motion in future airway flow studies. 

The literature review shows that although numerous numerical and experimental studies have been performed, very few of them adopt numerical and experimental methods simultaneously with dedicated validation arrangements. In a continuing effort to better understand human pharyngeal airway physiology, this study presents a comprehensive study of the airflow dynamics in a realistic human pharynx model based on a combined research approach that incorporates numerical modelling and particle image velocimetry (PIV) measurements. Intricate flow patterns including wall static pressure, streamlines and velocity distributions are analyzed in detail. 

## 2. Materials and Methods

### 2.1. Pharynx Model Reconstruction

A three-dimensional model was generated from CT scans of a healthy 25-year-old, non-smoking Asian male (170 cm height, 75 kg mass). The scanned data in the format of DICOM consists of *x*-*y*-*z* coordinates of the airway perimeters for cross-sections spaced at intervals of 1 mm and spanned 83 mm of the airway from the nasopharynx bend to the pharynx. 

**Figure 2:**
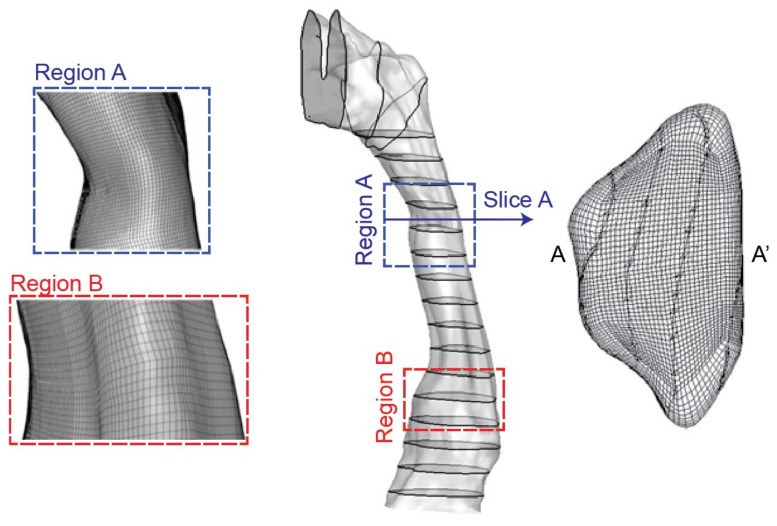
Computational pharyngeal model with cross-section slices and mesh.

Then the DICOM files from the scanned images were segmented using a commercial software MIMICS (Materialise, Leuven, Belgium). Then the airway lumen was obtained through creating a surface wrapping around the airway segment. Finally, the pharyngeal airway model was saved as STL file for 3D printing of cast replica and numerical mesh generation. To facilitate the fabrication process of the airway replica, the epiglottis feature at the narrowest section of the airway (Region B in Figure 2) was smoothed out in the present airway model. Further detailed information regarding the scan protocol and its reconstruction into a usable 3D geometry model can be found in literature [22,23].

### 2.2. Numerical Setup

The pharyngeal airway mode saved in STL format was imported into ICEM-CFD (ANSYS, Canonsburg, PA, USA) for mesh generation. A structured mesh was created with a refined distribution in the near wall region to account for any sharp flow gradients. In regions where velocity remains almost constant, relatively coarser mesh was employed. Then, an initial mesh of 650,000 cells was created. Mesh independence testing based on five mesh configurations (i.e., 0.65, 0.8, 1.1, 1.4, 1.8 million) were also performed. Through comparing velocity predictions along with a prescribed line location in the airway (line 4 in Figure 6), a final pharyngeal model containing 1.4 million mesh elements was adopted for numerical simulations (Figure 2). To establish fully developed airflow fields, artificial airway extensions were added at the inlet and outlet of the pharyngeal airway model for both numerical and experimental setups.

In this study, only light breathing condition is considered, and the airflow is assumed to be steady laminar flow with a constant inhalation flow rate of 10 L/min [24,25]. Previous numerical studies using a range of turbulence models such as *k* – *ε*, *k* - *ω* and the *v*^2^ - *f* model [26,27,28] showed that the turbulent kinetic energy and its production was extremely low at comparable inhalation conditions. Therefore, in present study, the turbulence influence was not considered. 

The commercial CFD code, ANSYS-FLUENT v18.0, was used to solve the fluid flow equations. Mass flow inlet condition and outflow condition were used for the CFD model, all wall surfaces were set as no-slip wall condition. The governing transport equations were discretized using the finite-volume approach. The QUICK scheme was used to approximate the convective terms while second order accurate central differencing scheme is adopted for the diffusion terms. The pressure-velocity coupling used the SIMPLE method, and the convergence was able to be reached at a criterion of 1 × 10^−7^.

### 2.3. Experimental Setup

**Figure 3:**
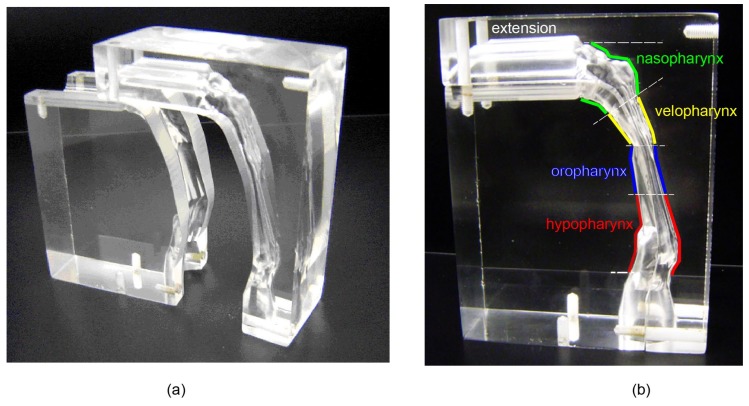
The cast replica of the human pharyngeal airway, (**a**) dismantled blocks (**b**) assembled model labelled with anatomical terminology.

As shown in Figure 3, a real sized 3D cast replica was manufactured using 3D printing technique. Due to the manufacturing difficulty of creating a hollow flow passage in an acrylic block, the whole model was carved into two half blocks (Figure 3a). Then, the two blocks were joined together by glue and screws (Figure 3b).

The experimental flow circuit comprised of the physical model, a fluid tank, and a suction pump. The model was submerged in a tank and connected to a pump that controls the flow rate. The index of refraction of the working fluid was matched to the acrylic model to eliminate reflection of the laser sheet. This required a mixture of water and glycerol (20:80 in weight, *υ* = 5.13 × 10^−5^ m^2^/s at 25 °C). The mixture density is 1.208 g/cm^3^, which is 1000 times of air density (1.2 × 10^−3^ g/cm^3^). The kinematic viscosity of air is *υ* = 1.565 × 10^−5^ m^2^/s at 25 °C. 

For inhaled air at *Q*_air_ = 10 L/min, the experiment flow should meet the same Reynolds number at the same location:

(1) Reair= Reexp

(2) $$\frac{\rho_{\mathrm{air}}\times Velocity{\;}_{\mathrm{air}}\times D}{1.565\times 10^{-5}}=\frac{\rho_{mix}\times Velocity{\;}_{\mathrm{mix}}\times D}{5.13\times 10^{-5}}$$

(3) $$\frac{\rho_{\mathrm{air}}\times Q{\;}_{\mathrm{air}}}{1.565\times 10^{-5}}=\frac{\rho_{mix}\times Q{\;}_{\mathrm{mix}}}{5.13\times 10^{-5}}$$

(4) $$Q{\;}_{\mathrm{mix}}=\frac{5.13\times 10^{-5}}{1.565\times 10^{-5}}\times \frac{\rho_{air}}{\rho_{mix}}\times Q{\;}_{\mathrm{air}}=3.23\times 10^{-3}\times Q{\;}_{\mathrm{air}}$$

**Figure 4:**
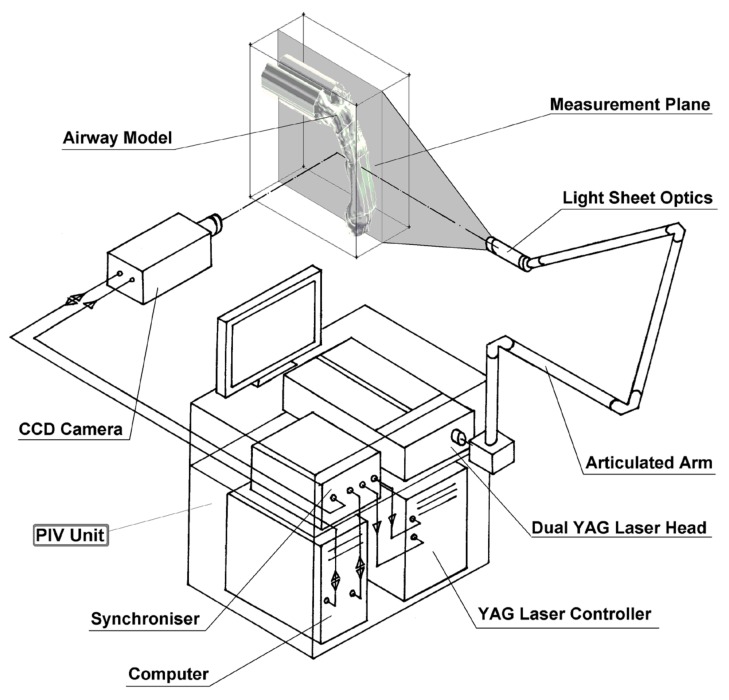
Schematic diagram of the PIV setup for airflow measurements.

Therefore, for the water-glycerol mixture, the flow rate is *Q* = 3.23 × 10^−2^ L/min. The flow field within the airway was measured using 2D PIV (Figure 4). The PIV system consists of a 1.3 Megapixel (1280 × 1024 pixels) 12-bit digital CCD camera (PCO Imaging, Kelheim, Germany) which was synchronized with a 120 mJ double-cavity Nd:YAG laser (New Wave, Fremont, CA, USA). The laser beam is expanded by a cylindrical lens to form a 2 mm thick plane vertical light sheet directly cutting through the airway. The laser head is supported on a horizontal traverse unit (+/- 0.017 mm), allowing the measurement plane to be moved by a repeatable and quantifiable amount. Measurements are taken in 3 mm slices starting from the mid-sagittal plane. The field of view of the CCD camera is 165 × 132 mm^2^ using 1280 × 1028 pixels of the CCD array. The smallest resolvable length scale of the PIV set-up, which is the real length of each pixel, equals 128.9 μm. The seeding particles are TSI silver-coated hollow glass beads with mean diameter of 14 μm and relative density of 1.65 to air, which is large enough to create light reflections for measurement with negligible disturbance to the flow streamlines.

## 3. Results and Discussion

### 3.1. Flow Patterns Comparison

**Figure 5:**
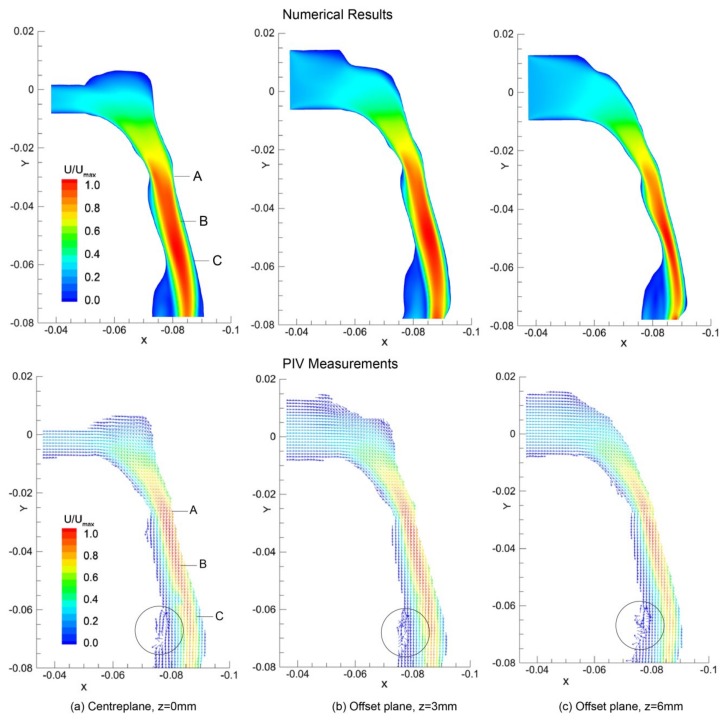
Velocity contours (CFD) and Velocity vectors (PIV) plot in the sagittal planes at (**a**) *z* = 0 mm (**b**) offset *z* = 3 mm plane, and (**c**) offset *z* = 6 mm plane, and normalised by the global maximum velocity. The plot axis units are in metres. Vectors are uniform in length to emphasis regions of recirculation, highlighted with circles. Landmark locations include: (A)-minimum cross-sectional area in the velopharynx; (B)-oral pharyngeal junction; and (C)-epiglottis tip.

General flow patterns in terms of velocity contours and vectors predicted by numerical simulation and PIV measurements are compared in Figure 5. The results were obtained from three *x* - *y* sagittal planes cutting through *z* = 0 mm, 3 mm, and 6 mm. 

In general, both CFD modelling and PIV measurements can predict the airflow patterns in the pharyngeal airway with a good agreement. Acceleration is found after the nasopharynx bend with high velocities in the posterior pharyngeal wall. The minimum cross-sectional area in the velopharynx (labelled as A in Figure 5) is found at *y* = −0.028 m having an area of 1.20 cm^2^. This cross-sectional area reduction produces a jet-like flow as the inhaled air squeezes through. Meanwhile, flow separation occurs at the anterior section of the airway. Further downstream an additional but larger recirculation is formed downstream of the narrowest site of the airway at the level of epiglottis (label C). These separation points and large flow gradients give rise to high susceptibility for airway collapse since it correlates with sharp pressure gradients [29,30]. However, more detailed flow features such as secondary flow vortices could not be revealed here, but it will be shown numerically later. 

### 3.2. Local Velocity Comparison

**Figure 6:**
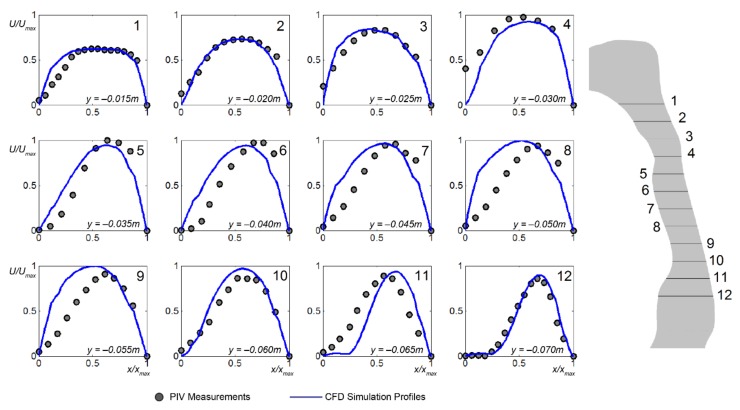
Horizontal normalised velocity profiles at different heights (*y*-axis in Figure 5a) in mid-sagittal plane *z* = 0 mm). The velocity magnitudes, *U* are normalised by its global maximum value, *U*_max_, and the airway lumen distance *x* from the anterior wall to the posterior wall is normalised by *x*_max_.

Twelve velocity profiles at different *y*-distance along the mid-sagittal plane, *z* = 0 mm were extracted from the CFD simulations and compared with the PIV measurements (Figure 6). The first line profile occurs at *y* = −0.015 m just after the nasopharynx bend with each subsequent line profile taken at 5 mm increments. 

Mainly, the computational results can predict the flow profiles well particularly in the immediate region past the nasopharynx bend (*y* = −0.015 m to *y* = −0.035 m). The peak velocities are closely matched with the PIV measurements. However, notable discrepancies were found near anterior wall in further downstream locations (*y* = −0.040 m to *y* = −0.065 m), where the current CFD modelling approach over predicts the near wall velocity. This may attribute to the steady laminar flow assumption used in the current CFD model, which limits the numerical simulation capacity to resolve the unsteady flow eddies occurring at this region.

The main velocity profile characteristics can be described by the following: (a) the velocity profiles became skewed in the curved portion of the pharynx due to centrifugal forces, and (b) flow separation occurred at abrupt geometrical changes where the cross-sectional area decrease and then increased where a jet stream is formed. The computed profiles tend to over-predict on the anterior wall side, not producing skewed profiles as that found in the experimental measurements. At the epiglottis low velocities and flow recirculation prevail, represented in the profiles (*y* = −0.065 m, and *y* = −0.070 m) with a near-zero velocity. Such profiles lead to an entrainment of fluid and possible build-up of any transported particulates that reaches this region.

Despite discrepancies exist between the current numerical results and PIV measurements, general airflow features (i.e., flow acceleration, separation) are confirmed by both methods. Due to the restrictions imposed by the phantom geometry, current PIV measurement setup could not provide cross-sectional velocity contours as it requires much more efforts to adjust the CCD camera and laser sheet positions. To explore more fluid mechanics in human pharyngeal airway, additional numerical simulation results are provided.

### 3.3. Streamlines and Cross-Sectional Velocity Contours

**Figure 7:**
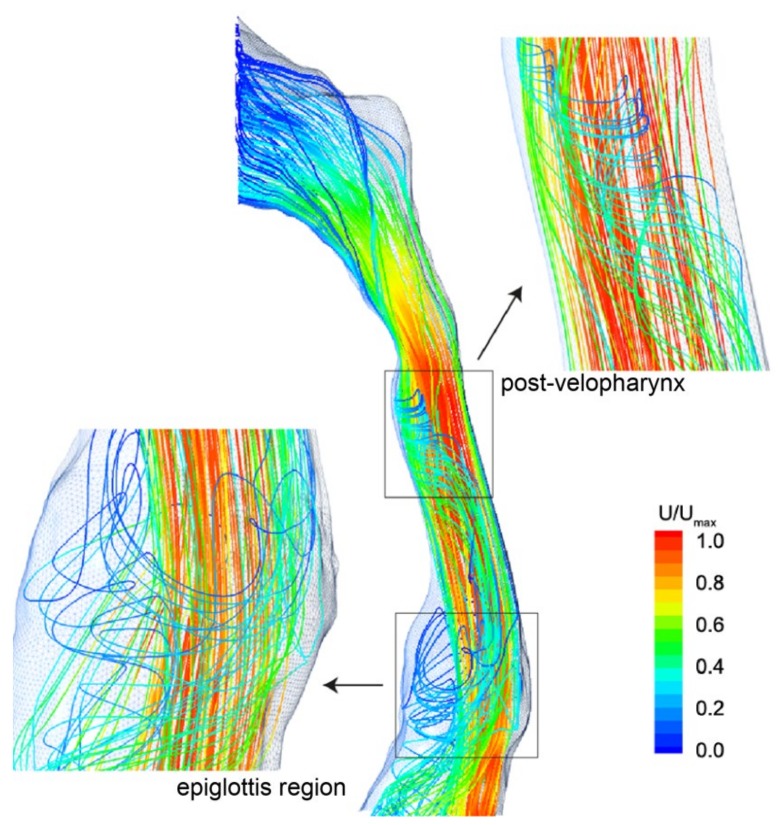
Streamlines coloured by velocity magnitude, highlighting the recirculating flow regions occurring downstream of the flow separation points.

Streamlines with the recirculating secondary flows are shown in Figure 7. Similar to the velocity contours depicted in Figure 5, flow acceleration is found as a jet like stream squeezes through the velopharynx, while the flow separation due to the abrupt airway changes produces swirling streamlines around the main bulk flow in the oropharynx region. Further downstream in the epiglottis, a similar but larger swirl pattern is formed. This coincides with the presence of the low velocity which surrounds the main center fluid flow.

**Figure 8:**
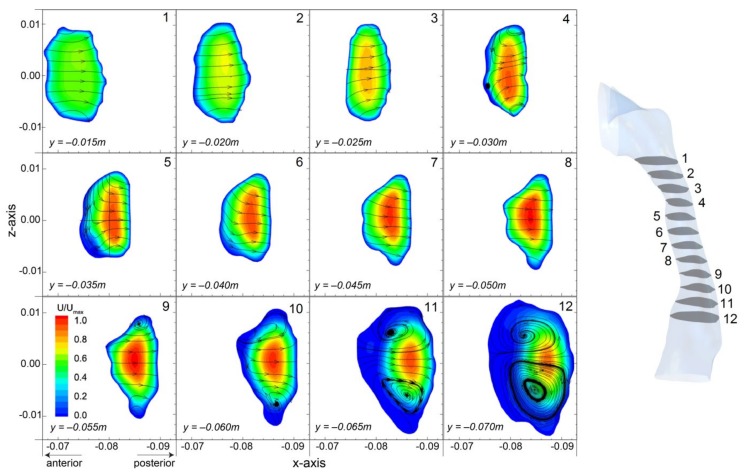
Velocity contours of axial velocity in the *y*-direction overlaid with cross-flow streamlines (*x*- and *z*- velocity components) in cross-sectional planes at the same *y*-heights used for the velocity profile comparisons in Figure 6. Each plane is relative to each other in size and location. Velocities are normalised by the global maximum.

Velocity contours of 12 cross-sectional plans at different *y*-heights are shown in Figure 8. Each slice is sized and displayed in its relative position allowing direct geometry comparisons. Observations of the geometry itself highlights the reduction in cross-sectional area, and the minimum plane occurs at plane 4 (*y* = −0.030 m). The results show the geometry curves posteriorly since the location of each cross-sectional slice shifts posteriorly. At the beginning, evenly distributed axial flow is found at plane 1 (*y* = −0.015 m). Then, airflow develops with increased core velocity in the center as the cross-sectional area decreases to plane 4 (*y* = −0.030 m). Further downstream, the bulk flow remains in the center of the airway (plane 5 to 9) and then shifts posteriorly due to the enlarged airway geometry (plane 10 to 12).

In addition, secondary flow patterns illustrating by streamtrace lines and flow directions are provided. It is found that the airflow is directed posteriorly due to the nasopharynx bend and the posterior progression of the pharyngeal airway. A small recirculating vortex is found near the anterior wall at plane 4 (*y* = −0.030 m), and the streamtrace lines coming from the sides and bending back out. Downstream the flow recovers with the streamtraces directed posteriorly, relatively linear and remains consistent down to plane 8 (*y* = −0.055 m), where the cross-sectional plane starts to expand. Further downstream, a characteristic pair of counter-rotating swirling vortexes caused by the axial flow pushing effect towards the posterior wall. The presence of these secondary flow vortexes indicates enhanced mixing in the cross-section and may contribute into improved residence times of inhaled particles.

The cross-sectional slices also reveal the antero-posterior to transverse (*AP*/*T*) diameter ratio is in the range of 0.4–0.7 which fits with data for a healthy pharyngeal subject where *AP*/*T* < 1.0 [2,31]. It should be noted that the airway geometry in this study was obtained from CT-scans during wakefulness where the upper airway dilator muscles are activated, and these measurements would differ during sleep.

### 3.4. Pressure and Flow Resistance

**Figure 9:**
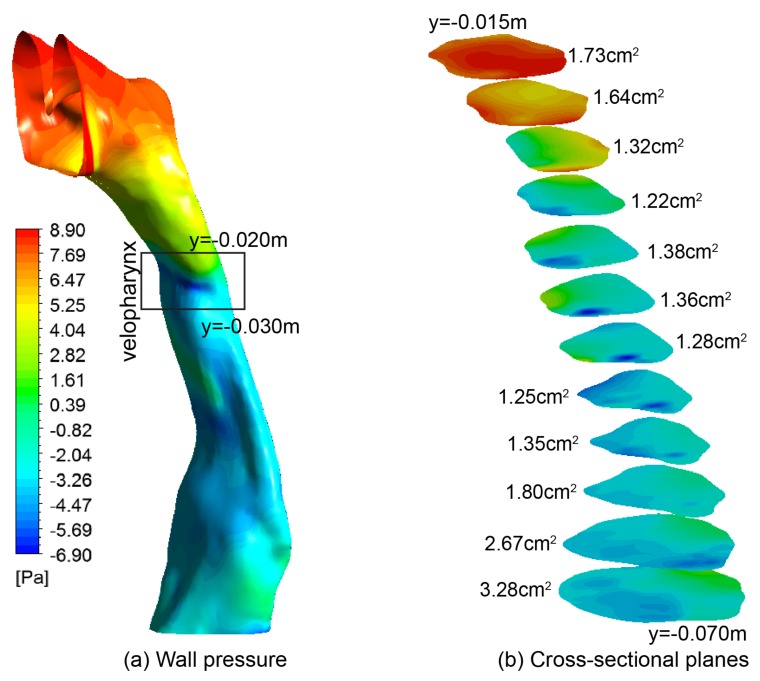
(**a**) Pressure contours at the wall; (**b**) Pressure contours at 12 cross-sectional slices. The cross-sectional area is given in cm^2^ for each slice.

The pressure distributions over the airway surface and 12 selected cross-sectional planes are plotted in Figure 9. Two regions of pressure gradients separated by the velo-oropharynx junction are observed. Above the velo-oropharynx neck, a rapid pressure drop is found, while below the velo-oropharynx neck, pressure drop is much more gradual. This is attributed to the velopharynx acting as a converging-diverging nozzle, which accelerates and then decelerates the airflow. Then in the epiglottis, the airway volume expands gradually. A minimum pressure with value of −6.90 Pa is established at the throat of the nozzle-shaped airway. This negative pressure field greatly contributes towards the collapse of the soft palate inducing obstructive sleep apnea. In addition, in-plane pressure variation between the anterior and posterior walls is examined for each slice (Figure 9b). Especially, for the velo- and oro-pharynx, abrupt pressure variation is found in local regions, where low pressure regions are surrounded by higher pressure. Consequently, greater fluid dynamic forces in influencing the surrounding pharyngeal walls are created.

**Figure 10:**
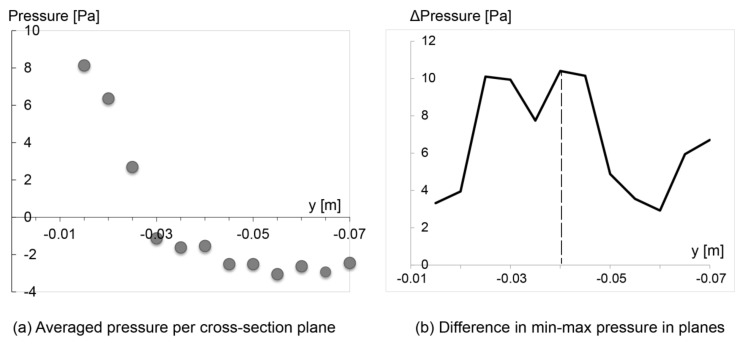
(**a**) Pressure drop of the main airflow; (**b**) Pressure difference between the minimum and maximum pressure values within each slice.

Airway resistance is often a preferred variable when analyzing breathing flows [15], and it is directly linked to pressure drop. To contribute more insights into the pharyngeal compliance, quantitative pressure drop analysis along the pharyngeal airway is provided (Figure 10). 

From Figure 10a, a sharp pressure decrease between the pre- and post- velopharynx is found, where pressure decreases by 9 Pa over a distance of 0.015 m in pre-velopharynx region, while for post-velopharynx, the pressure drop slows down dramatically with a 2 Pa reduction over a distance of 0.04 m. This suggests greater flow resistance in the pre-velopharynx region. Figure 10b depicts the pressure difference between the minimum and maximum pressure values within each slice, where the largest variation occurs at plane 5 (*y* = −0.035 m) in the oropharynx region. This is consistent with the production of the venturi effect in the velo-oropharynx region that increases the flow velocity. 

It should be noted that the anterior wall of the velopharynx and oropharynx is composed primarily of the soft palate, tongue and lingual tonsils. The posterior wall is bounded by a muscular wall, while the lateral pharyngeal wall is a complex composition of muscle, lymphoid tissue, and pharyngeal mucosa. In combination, considering the venturi effect, the surrounding tissue composition and their biomechanical properties, strong possibility of airway constriction by the collapse of the tongue and soft palate onto the posterior muscular wall is anticipated [29,30,32]. 

## 4. Discussion and Limitations

The current numerical simulations and experimental measurements in the present pharyngeal airway model revealed the three-dimensional nature of the airflow and the corresponding pressure distribution on the airway wall. This enables us to quantify the interaction between airway anatomy and airway dynamics. Despite the research topic has been frequently studied, research findings from previous research attempts are often not replicable due to their subject-specific nature, and thus the impact of inter-subject variability on obtained flow fields often poses an obstacle for relevant researchers in adopting these data for validation purposes. As a response to this research need, this paper aims to remove this barrier by developing an open accessed benchmark model that allows other researchers in establishing physiologically reasonable flow fields and validating their own numerical models with great ease. In addition, to maximize the data applicability for other airway models at similar or different spatial scales, PIV measured flow velocities were converted into dimensionless values by dividing the local velocity with maximum velocity in the flow field, which will be convenient for researchers and knowledge users in applying the research findings into their own projects. To promote research collaboration in this field, the authors agreed to make this developed model and related research findings available to the general public (all model enquiries subject to email communication with the corresponding author). This work expects to serve as a benchmark study for other researchers in obtaining validated numerical models at significantly reduced cost. The airway models and revealed flow fields reported in this paper lay a scientific underpinning for future investigations in dealing with the interactions between soft tissue movement and respiratory airflow. 

This study was confined to a single approximate airway model under steady-state flow condition. Future studies may include unsteady respiratory flow conditions to analyse the effects of transient flow on airflow dynamics. PIV measurements from a fully detailed airway remain challenging due to demanding resolution requirements and the need to avoid optical distortion, which becomes increasingly challenging with significantly deformed airways. To provide a viable solution, the present study has demonstrated a manufacturing and measurement feasible friendly pharyngeal airway model, which can be potentially utilized in other relevant research studies. 

Further experimental work to validate the flow field for higher inhalation condition (such as 50 L/min during or after intense exercise) is also necessary where the flow is turbulent so as to further study the effectiveness of widely adopted turbulent flow modelling approach − the Reynolds-averaged Navier–Stokes equations (RANS) based turbulent models.

## 5. Conclusions

Knowledge of airflow characteristics in pharynx is essential to understand the physiological and pathological aspects of obstructive sleep apnea. This paper presents a numerical and experimental study of the airflow in the pharynx. The general good agreements between numerical and experimental results confirm the existence of the main airflow characteristics within the pharyngeal airway, in which a rich variety of fluid dynamic characteristics is revealed. This includes: (i) flow separation in the velopharynx and epiglottis where jet flows occur; (ii) recirculation in the anterior wall, and the presence of secondary flow vortexes; (iii) greatest pressure drop and hence resistance is found in the superior half of the airway; (iv) large pressure variation in the transverse plane occurs at the velo-oropharynx junction, which is where common airway collapse occurs. These results are consistent with previous research findings, the fluid dynamics reported here provide important insights for clinical researchers in better interpreting the physiology and pathology of obstructive sleep apnea. Despite discrepancies exist between the current CFD model and PIV measurements, more sophisticated modelling approach such as large eddy simulation can be applied to further improve the numerical simulation accuracy. However, this will require more modelling efforts and computational resources, which will significantly lower the research efficiency. 

In summary, intricate airflow characteristic are studied by numerical modelling and experimental measurement approaches in this study. The experimental data can contribute towards a database of validation for CFD simulations. The research methods prosed by this paper can provide insights and contribute towards improved treatments of the obstructive sleep apnea.
